# Effects of Selenium Application on Fermentation Quality, Chemical Composition, and Bacterial Community of Hybrid *Pennisetum* Silage

**DOI:** 10.3390/microorganisms12112144

**Published:** 2024-10-25

**Authors:** Xinzhu Chen, Shuiling Qiu, Liang Huang, Yanie Yang, Xiaoyun Huang, Xiusheng Huang, Deqing Feng

**Affiliations:** 1 Institute of Resources, Environment and Soil Fertilizer/Institute of Animal Husbandry and Veterinary Medicine, Fujian Academy of Agricultural Sciences, Fuzhou 350013, China; xinzhuchen1985@163.com (X.C.); shuilingqiu@126.com (S.Q.); mandyhwang96@163.com (L.H.); yanieyang1992@163.com (Y.Y.); huangxy364@163.com (X.H.); 2 College of Animal Sciences (College of Bee Science), Fujian Agriculture and Forestry University, Fuzhou 350002, China; 3 Fujian Key Laboratory of Animal Genetics and Breeding, Fuzhou 350013, China; 4 Fujian Engineering and Technology Research Center for Recycling Agriculture in Hilly Areas, Fuzhou 350013, China

**Keywords:** selenium, silage, bacterial community, fermentation quality, hybrid *Pennisetum*

## Abstract

The primary objective of this study is to facilitate the conversion of inorganic selenium (Se) into organic Se within plants via assimilation, subsequently feeding it to livestock and poultry to enhance healthy animal production and yield Se-enriched livestock and poultry products. Therefore, it is imperative to first investigate the impact of varying Se doses on the agronomic traits of plants as well as their forage storage and processing. This experiment investigated the effect of Se fertilizer application on the fermentation quality, chemical composition, and bacterial community of *Pennisetum americanum* × *Pennisetum purpureum* cv Minmu 7 (HPM7). There were nine Se fertilizer dissolution levels of HPM7 treated, which were 0 mg/kg (Se0), 0.50 mg/kg (Se1), 1.00 mg/kg (Se2), 2.00 mg/kg (Se3), 5.00 mg/kg (Se4), 10.00 mg/kg (Se5), 20.00 mg/kg (Se6), 30.00 mg/kg (Se7), 40.00 mg/kg (Se8), and 50.00 mg/kg (Se9). The results showed that after silage, the water-soluble carbohydrates of Se1, Se2, and Se3 were lower than Se0, and the pH of Se3, Se4, and Se6 were lower than the Se0. The number of OTUs in the nine groups was sequentially Se1 > Se2 > Se3 > Se8 > Se6 > Se5 > Se7 > Se4 > Se0. The dominant bacterial phyla in silage samples were Firmicutes and Proteobacteria. Compared with Se0, Bacterial Shannon index in Se1 and Se2 were higher, while Chao1 and ACE indices of Se1, Se2, Se3, Se5, and Se6 were higher. A beta diversity analysis indicated that the Se1 exhibited the highest number of significant biomarkers. *Escherichia coli* between Se0 and Se3 and *Clostridium sardiniense* and *Clostridium perfringens* between Se0 and Se1 exhibited significant differences at a species level. The most abundant pathways for metabolism were membrane transport, carbohydrate metabolism, translation, replication, repair, and amino acid metabolism. The correlation analysis indicated that the dry matter content was negatively correlated with *Bacillus* (*p* < 0.01), *Lactobacillus* (*p* < 0.05), *Pediococcus* (*p* < 0.05), and *Hirschia* (*p* < 0.05); the contents of neutral detergent fiber and hemi-cellulose were positively correlated with *Lactobacillus* (*p* < 0.05, *p* < 0.01). The protein content was negatively correlated with *proteus* (*p* < 0.05). This study demonstrated that the application of Se fertilizer could enhance the Se content in HPM7. The optimal fertilization concentration was found to range from 0.50 to 2.00 mg/kg, which facilitates the metabolism of soluble carbohydrates and enhances both the fermentation quality and microbial relative abundance of HPM7 silage.

## 1. Introduction

Hybrid *Pennisetum* (*Pennisetum americanum* × *Pennisetum purpureum*, HP) is the dominant perennial herb in tropical and subtropical areas because of its rapid growth, high biological yield, and good adaptability to the environment, especially in the south of China [1,2]. It also has developed roots, most of which have erect stems, tillering ability, rich nutrition, more balanced amino acid content, and other characteristics [3]. With the increasing demand for forage, more and more studies have been conducted on its preservation and valuation [4,5].

The HP, which is planted in the south and other wet and rainy areas, is difficult for hay modulation. Silage, which is fermented in an anaerobic environment by lactic acid bacteria (LAB) to convert soluble sugars into lactic acid (LA)-based organic acids, is effective in preserving the nutrients and increasing the palatability and feed utilization ratio of grasses such as HP [6]. However, it is difficult to produce excellent silage with HP because of its high buffering capacity (BC), low water-soluble carbohydrate (WSC), and lack of LABs [7]. Therefore, special treatment is needed for silage modulation.

Selenium (Se) is an essential trace element in animals, playing crucial physiological roles such as participating in antioxidant reactions, regulating immune function, and maintaining cardiovascular health [8]. Livestock, poultry, and humans with severe Se deficiency often suffer from various diseases because they cannot obtain enough Se from local food [9]. Studies have shown that fertilization can effectively increase the accumulation of Se in forage grass, and through the migration and transformation in the grass and animal food chain, it is an important method for effective supplementation [10]. Currently, studies on Se enrichment enhancement of alfalfa, ryegrass, oat, milkvetch, white clover, and other forage grasses have been carried out [11,12,13,14,15]. However, there are few reports on the Se enrichment of *Pennisetum* forages. Therefore, we hypothesized that the appropriate level of Se fertilizer application will improve the fermentation quality and chemical composition and enhance the microorganism relative abundance of silages. The objective of this study is to investigate the effects of Se application on the fermentation quality, chemical composition, and bacterial community of hybrid *Pennisetum* silage. And then, the Se-enriched plant is utilized as feed for livestock and poultry to enhance their antioxidant capacity, immune function, reproductive performance, and other physiological traits, ultimately providing healthy and safe Se-rich food sources for human consumption.

## 2. Materials and Methods

### 2.1. Raw Materials

Materials: Hybrid *Pennisetum* (*Pennisetum americanum* × *Pennisetum purpureum* cv Minmu 7, HPM7) were provided by the Institute of Agricultural Ecology, Fujian Academy of Agricultural Sciences. Stem cuttings were used to transplant long seedlings on 15 June 2021.

Se solution: Sodium disodium selenite (Na_2_SeO_3_) (analytical reagent (AR), Se content of 45.50%) was used as a Se source. According to the experimental design, the Se content needed for each treatment was accurately calculated, weighed, and dissolved in 50 mL of distilled water for use.

Soil: The basic physical and chemical properties of red soil were pH 7.80, organic matter 1.70 g/kg, total N 0.01 g/kg, alkaline N 7.76 mg/kg, available P 0.70 mg/kg, available K 70.80 mg/kg, and total Se 0.07 mg/kg.

Basin: Gallon basin, 20 cm diameter, 25 cm height.

### 2.2. Base Conditions

The HPM7s were planted in Quantou Experimental Base (Fuzhou, China) at 26°130 N and 119°340 E at an altitude of 601 m above sea level. The average annual temperature, the mean annual rainfall, the average annual sunshine hours, the relative humidity, and the average wind speed of the area are 21.2 °C, 1495.8 mm, 1989.3 h, 66%, and 1.5 m/s, respectively [16].

### 2.3. Test Design and Treated Method

There were nine Se fertilizer dissolution levels of HPM7 treated, which were 0 mg/kg (Se0), 0.50 mg/kg (Se1), 1.00 mg/kg (Se2), 2.00 mg/kg (Se3), 5.00 mg/kg (Se4), 10.00 mg/kg (Se5), 20.00 mg/kg (Se6), 30.00 mg/kg (Se7), 40.00 mg/kg (Se8), and 50.00 mg/kg (Se9).

The 15.0 kg of red soil was sprayed with the prepared Se solution evenly then activated for 7 days after applying 15 g/pot base fertilizer. The N, P, and K compound fertilizer was used as base fertilizer, its ratio of N, P_2_O_5_, K_2_O was 15:15:15. The HPM7 was transplanted on 15 June 2021, with one plant per pot. Seven replicates for each treatment by random block arrangement.

### 2.4. Silage Making

The HPM7s were manually harvested at approximately 3 m high on 15 September 2021. After wilting for a certain time under natural conditions, the materials were cut 2–3 cm and packed into plastic bags (30 cm × 20 cm, Mingkang Packing Co., Ltd., Zhongshan, China). There were four replicates of each Se fertilizer solubility level treatment. The plastic bags were sealed with a vacuum sealer (SINBO Vacuum Sealer; Hong Tai Home Electrical Appliance Co., Ltd., Hong Kong, China) and kept in the laboratory at ambient temperature for 60 days.

### 2.5. Chemical Analysis

The dry matter (DM) content was determined after drying in a 68 °C oven for 48 h [17]. Crude protein (CP) and ammonia-N concentrations were analyzed according to AOAC methods [10]. Ether-extract content (EE) was determined using a soxhlet extraction method (Ankom XT10i, New York, NY, USA). Crude ash (CA) was determined using a dry ashing method [10]. Water-soluble carbohydrates (WSCs) were used in a anthrone reaction rate assay [18]. Neutral detergent fiber (NDF) and acid detergent fiber (ADF) contents were measured according to a previous procedure [19]. Hemi-cellulose (HC) content was calculated as follows: HC = NDF − ADF.

### 2.6. Fermentation Quality Analysis

For the analysis of silage quality, a 20 g sample was mixed with 80 mL of distilled water and placed at 4 °C for 18 h. Then, the material was filtered, and the filtrate was used to measure pH, ammonia-N, and organic acids. pH was measured with a pH meter (PHS-3B; Hangzhou Chincan Trading Co., Ltd., Shanghai, China) [5]. Organic acid contents were analyzed by high-performance liquid chromatography (Sodex RS Pak KC-811column; Showa Denko K.K., Kawasaki, Japan; DAD detector: 210 nm, SPD-20A, Shimadzu Co., Ltd., Kyoto, Japan; eluent: 3 mm HClO_4_, 1.0 mL/min; temperature: 40 °C).

### 2.7. Microbial Diversity Analysis

After each bag of silage sample was mixed evenly, FMs of 10 g samples were transferred aseptically using flame-sterilized forceps and placed into sterilized washing buffer (0.1 M potassium phosphate buffer, pH 7.0) and sonicated (frequency: 40 kHz) for 5 min in an ultrasonic cleaning bath, followed by vigorous shaking of the samples for 20 min until microorganisms from samples floated into the buffer at ambient temperature [20].

Using a pipette to take exactly 1 mL of buffer solution for bacteria, LAB, yeasts, and molds, counting serially, they were diluted in a sterile saline solution (0.85% NaCl). Another buffer solution was used for total microorganisms recovered by centrifugation at 8000× *g* for 20 min at 4 °C. Total microorganisms DNA from the recovered epiphytic cells was extracted according to the method described by Chen et al. [20].

#### 2.7.1. Microbial Count Analysis

LABs were counted on an MRS agar after incubation in an anaerobic incubator (N_2_:H_2_:CO_2_ = 85:5:10; YQX-II, CIMO Medical Instrument Manufacturing Co. Ltd., Shanghai, China) at 37 °C for 3 days. Aerobic bacteria were counted on a nutrient agar (Nissui-seiyaku Ltd., Tokyo, Japan), and yeasts and molds were counted on potato dextrose agar (Nissui-seiyaku Ltd.) acidified with sterilized tartaric acid solution (10%) to pH 3.5. The agar plates were incubated under aerobic conditions at 37 °C for 3 days. A statistical analysis was conducted on the data of the fermentation products and chemical composition of the silages.

#### 2.7.2. DNA Extraction, PCR Amplification, and High-Throughput Sequencing

DNA total genome DNA from samples was extracted using CTAB/SDS method [20]. DNA concentration and purity were monitored on 1% agarose gels. According to the concentration, DNA was diluted to 1 ug/μL using sterile water. Amplicon Generation 16S rRNA genes of distinct regions (V3-V4/16S) were amplified using a specific primer (515F and 806R) with the barcode. All PCR reactions were carried out with 15 μL of Phusion^®^ High-Fidelity PCR Master Mix (New England Biolabs, Ipswich, MA, USA); 0.2 μM of forward and reverse primers, and about 10 ng template DNA, were used. Thermal cycling consisted of initial denaturation at 98 °C for 1 min, followed by 30 cycles of denaturation at 98 °C for 10 s, annealing at 50 °C for 30 s, and elongation at 72 °C for 30 s. Finally, 72 °C for 5 min. Mix the same volume of IX loading buffer (contained SYB green) with PCR products and operate electrophoresis on 2% agarose gel for detection. PCR products were mixed in equidensity ratios. Then, the mixture of PCR products was purified with Qiagen Gel Extraction Kit (Qiagen, Germany). Then, the samples were sent to Novogene Biology Ltd. (Beijing, China) for 16s rDNA high-throughput sequencing analysis. Sequencing libraries were generated using TruSeq^®^ DNA PCR-Free Sample Preparation Kit (Illumina, San Diego, CA, USA) following the manufacturer’s recommendations, and index codes were added. The library quality was assessed on a Qubit@2.0 Fluorometer (Thermo Scientific, Waltham, MA, USA) and Agilent Bioanalyzer 2100 system. At last, the library was sequenced on an Illumina NovaSeq platform, and 250 bp paired-end reads were generated.

### 2.8. Statistics Analysis

Data on the chemical composition and microbial number were processed and analyzed with Excel and SPSS software, using a one-way analysis of variance method. The means were then compared for significance via Duncan’s multiple range method at (*p* < 0.05).

Paired-end reads were assigned to samples based on their unique barcode and truncated by cutting off the barcode and primer sequence. Paired-end reads were merged using FLASH (VI.2.7, http://ccb.jhu.edu/software/FLASH/, accessed on 7 October 2021) [21], a very fast and accurate analysis tool, which was designed to merge paired-end reads when at least some of the reads overlap with the read generated from the opposite end of the same DNA fragment, and the splicing sequences were called raw tags. Quality filtering on the raw tags were performed under specific filtering conditions to obtain the high-quality clean tag according to the QIIME (V1.9.1, http://qiime.org/index-qiime1.html, accessed on 7 October 2021) using a quality-controlled process [22,23]. The tags were compared with the reference database (Silva database, https://www.arb-silva.de/, accessed on 7 October 2021) using a UCHIME algorithm (UCHIME Algorithm, http://www.drive5.com/usearch/manual/uchime_algo.html, accessed on 7 October 2021) [24] to detect chimera sequences, and then the chimera sequences were removed [25]. Then, the effective tags were finally obtained.

## 3. Results

### 3.1. The Characteristics of HPM7

As shown in Table 1, the seedlings of HPM7 in the Se9 group, which treated 50.00 mg/kg Se in soil, did not survive. Therefore, there were no data for this processing group. With the increase in Se fertilizer concentration, the Se content of HPM7 significantly increased. Compared with Se0, the Se content of the Se8 group increased by 11.28 mg/kg. Different Se fertilizer concentrations had a significant effect on DM, CP, WSC, NDF, ADF, HC, and EE (*p* < 0.05). There was no significant difference in ash content and the number of LAB, yeast, mold, and bacteria among the nine groups (*p* > 0.05). The DM contents of the Se7 and Se8 groups were the significantly highest among the groups (*p* < 0.05); the CP content of the Se1 group was significantly higher than the Se4, Se5, Se6, Se7, and Se8 groups’ (*p* < 0.05); the WSC contents of the Se3 and Se5 groups were significantly higher than the Se1, Se4, Se6, Se7, and Se8 groups’ (*p* < 0.05); the NDF contents of the Se8 group were significantly higher than the Se3, Se6, and Se7 groups’ (*p* < 0.05); the ADF contents of the Se3 group were significantly higher than the Se4, Se5, Se6, Se7, and Se8 groups’ (*p* < 0.05); the HC contents of the Se3 group were significantly lower than the Se4, Se5, Se7, and Se8 groups’ (*p* < 0.05); and the EE contents of the Se2 group were significantly higher than the Se8 group’s (*p* < 0.05).

### 3.2. Effect of Selenium on Chemical Composition and Fermentation Quality of HP Silages

The results of chemical composition and fermentation quality were shown in Table 2. Compared with the Se0 group, the DM contents of Se6, Se7, and Se8 groups; the CP content of the Se1 and Se2 groups; the WSC contents of the Se5, Se6, and Se7 groups; the lactic acid contents of the Se1, Se2, and Se4 groups; and the LAB numbers of the Se1, Se2, Se7, and Se8 groups were significantly higher (*p* < 0.05), while the DM contents of the Se3 group; the CP content of the Se4, Se5, Se7, and Se8 groups; the WSC contents of the Se1, Se2, and Se3 groups; the NDF contents of the Se8 group; the pH value of the Se3, Se4, and Se6 groups; the lactic acid contents of the Se7 and Se8 groups; the acetic acid contents of the Se4, Se5, Se6, Se7, and Se8 groups; and the butyric acid contents of the Se5, Se6, Se7, and Se8 groups were significantly lower (*p* < 0.05).

Furthermore, the DM contents of the Se7 and Se8 groups were significantly higher than the Se1, Se2, Se3, Se4, and Se5 groups’ (*p* < 0.05); the CP content of the Se1 group was significantly higher than the Se3, Se4, Se5, Se6, Se7, and Se8 groups’ (*p* < 0.05); the WSC contents of the Se5, Se6, and Se7 groups were significantly higher than the Se1, Se2, and Se3 groups’ (*p* < 0.05); the NDF contents of the Se8 group was significantly lower than the Se1, Se2, and Se4 groups’ (*p* < 0.05); the ADF contents of the Se1, Se4, and Se5 groups’ were significantly higher than the Se2 and Se8 groups’ (*p* < 0.05); the HC contents of the Se2 group was significantly lower than the Se3, Se5, Se6, Se7, and Se8 groups (*p* < 0.05); the pH values of the Se1, Se2, Se3, Se4, Se5, and Se6 groups were significantly higher than the Se7 and Se8 groups’ (*p* < 0.05); the lactic acid levels of the Se1 and Se2 groups were significantly higher than the Se4, Se7, and Se8 groups’ (*p* < 0.05); the acetic acid of the Se1, Se2, and Se3 groups were significantly higher than the Se4, Se5, Se6, Se7, and Se8 groups’ (*p* < 0.05); the propionic acid of the Se6 group was significantly higher than the Se5 and Se7 groups’ (*p* < 0.05); the butyric acid of the Se3 group was significantly higher than the Se5, Se6, Se7, and Se8 groups’ (*p* < 0.05); the LAB numbers of the Se2, Se7, and Se8 groups were significantly higher than the Se6 group’s (*p* < 0.05); and the yeast number of the Se5 was higher than the Se7 and Se8 groups’ (*p* < 0.05).

### 3.3. Effect of Selenium on Microbial Diversity of HPM7 Silages

#### 3.3.1. OUT Analysis in Different HPM7 Silages

**Figure 1:**
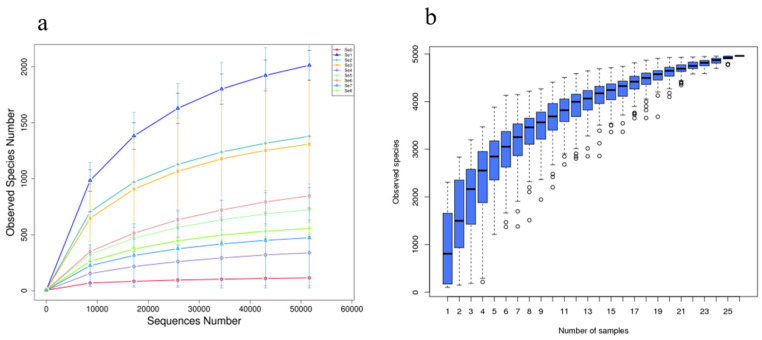
Sequences numbers (**a**) and observed species in different HPM7 silages (**b**). Note: Se0 to Se8 represent selenium fertilizer solubility levels of 0, 0.50 mg/kg, 1.00 mg/kg, 2.00 mg/kg, 5.00 mg/kg, 10.00 mg/kg, 20.00 mg/kg, 30.00 mg/kg, and 40.00 mg/kg, respectively.

After sequencing and quality control, an average of 86,653 valid data were obtained, with 69,645 effective data volume for quality control and 78.41% quality control effective rate. Clustering the sequence with 97% identity into OTUs (Operational Taxonomic Units) resulted in a total of 4984 OTUs in nine groups silages. When the sequencing number of observed spectra dilution curves reached 50,000 (Figure 1), the curves tended to flatten, and the Goods Coverage was greater than 0.99. This means that the sequencing data are reasonable and the sequencing depth is sufficient, which can basically reflect the diversity information of each group of microorganisms.

**Figure 2:**
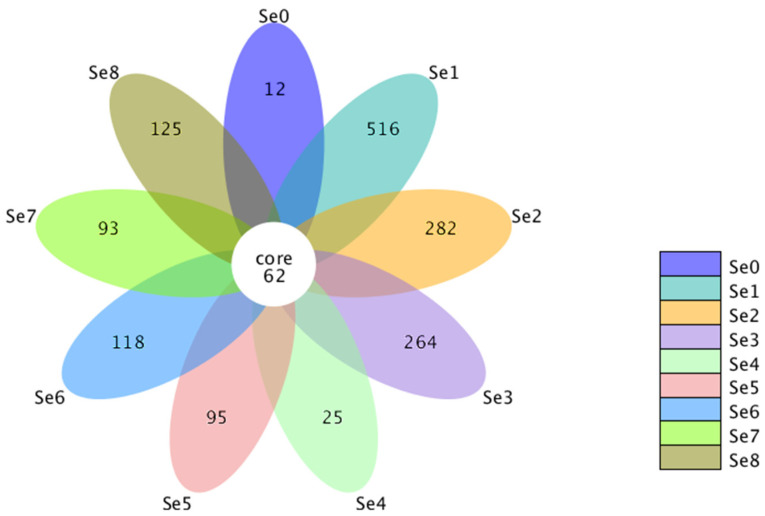
The Venn diagram of OTUs in different HPM7 silages. Note: Se0 to Se8 represent selenium fertilizer solubility levels of 0, 0.50 mg/kg, 1.00 mg/kg, 2.00 mg/kg, 5.00 mg/kg, 10.00 mg/kg, 20.00 mg/kg, 30.00 mg/kg, and 40.00 mg/kg, respectively.

The Venn plot reflected the common and unique OTUs among the sample groups (Figure 2). Compared with the Se0 group, the number of OTUs significantly increased by treated Se fertilizer. The number of OTUs in the nine groups was sequentially Se1 > Se2 > Se3 > Se8 > Se6 > Se5 > Se7 > Se4 > Se0. Among them, there were 62 core OTUs and 516, 282, 264, 25, 95, 118, 93, and 125 unique OTUs, respectively, for the Se1–8 groups.

#### 3.3.2. Bacterial Analysis in Different HPM7 Silages

**Figure 3:**
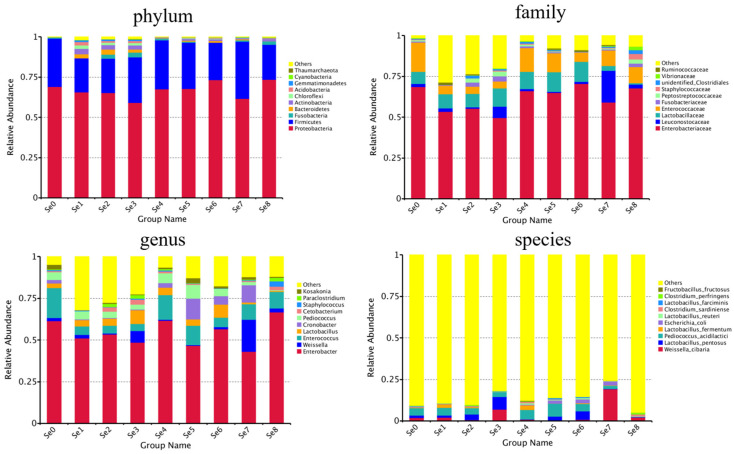
The relative abundance of microbial in HPM7 silages. Note: Se0 to Se8 represent selenium fertilizer solubility levels of 0, 0.50 mg/kg, 1.00 mg/kg, 2.00 mg/kg, 5.00 mg/kg, 10.00 mg/kg, 20.00 mg/kg, 30.00 mg/kg, and 40.00 mg/kg, respectively.

The relative abundance of dominant bacteria among the silages exhibited significant differences between Se0 and Se1–Se8 in Table 3 and Table 4, and Figure 3.

The dominant phyla, which had a relative abundance of more than 20%, were *Proteobacteria* (73.50–59.18%) and *Firmicutes* (35.53–21.01%) in the silages, while with a relative abundance of 1–20% were *Actinobacteria* (3.29%), *Bacteroidetes* (2.63%), *Acidobacteria* (2.14%), *Chloroflexi* (2.04%), and *Gemmatimonadetes* (1.22%) in the Se1 silage; *Bacteroidetes* (3.08%), *Actinobacteria* (2.79%), *Fusobacteria* (2.49%), *Chloroflexi* (1.47%), and *Gemmatimonadetes* (1.07%) in the Se2 silage; *Fusobacteria* (3.04%), *Actinobacteria* (2.50%), *Bacteroidetes* (1.98%), *Chloroflexi* (1.40%), and *Gemmatimonadetes* (1.29%) in the Se3 silage; *Fusobacteria* (1.06%) in the Se4 silage, *Actinobacteria* (1.05%) in Se5 silage; *Bacteroidetes* (1.09%) in the Se6 silage; and *Fusobacteria* (2.07%) and *Actinobacteria* (1.36%) in the Se8 silage. The relative abundance of Bacteroidetes in the Se2 group and Thaumarchaeota in Se3 group were significantly higher than in other groups, except the Se1 group (*p* < 0.05), in which Actinobacteria, Chloroflexi, Acidobacter, and Gemmatimonadetes were significantly higher than in the Se0, Se4, Se5, Se6, Se7, and Se8 groups (*p* < 0.05), Cyanobacteria in the Se8 group was significantly higher than in all the other groups except Se7 (*p* < 0.05) (Table 3).

At the family level, except for *Enterobacteriaceae*, which were the dominant in all the silages with a relative abundance of 49.76–70.46%, there were significant differences among silages which had a relative abundance above 10%, with *Enterococcaceae* (17.85%) in Se0, Others (28.59% and 23.46%) in Se1 and Se2, *Lactobacillaceae* (11.17%) and Others (19.96%) in Se3, *Lactobacillaceae* (10.48% and 12.20%) and *Enterococcaceae* (14.72% and 11.52%) in Se4 and Se5, *Lactobacillaceae* (12.35%) in Se6, *Leuconostocaceae* (19.26%) in Se7, and *Enterococcaceae* (10.01%) in Se8.

Except for Others, the dominant genus which had a relative abundance of more than 10% were *Enterobacter* (61.52%) and *Enteroccoccus* (17.85%) in Se0; *Enterobacter* (51.12%, 53.41%, 48.59%) in Se1, Se2, and Se3; *Enterobacter* (61.58%) and *Enteroccoccus* (14.71%) in Se4; *Enterobacter* (46.61%) and *Enteroccoccus* (11.52%) in Se5, *Enterobacter* (56.63%) in Se6; *Enterobacter* (43.05%), *Weissella* (19.23%), and *Cronobacter* (10.26%) in Se7; and *Enterobacter* (66.79%) and *Enteroccoccus* (10.01%) in Se8. The relative abundance of *Enterobacter* in the Se8 group was significantly higher than in the Se7 group (*p* < 0.05), *Weissella* in the Se7 group and *Staphylococcus* in Se8 group were significantly higher than that in other groups (*p* < 0.05), *Cronobacter* in Se5 group was significantly higher than that all other groups except the Se7 group (*p* < 0.05), *Pediococcus* in the Se5 group was significantly higher than that in Se3, Se7, and Se8 groups (*p* < 0.05), *Kosakonia* in the Se5 group was significantly higher than that in the Se1 group (*p* < 0.05) (Table 4).

At the species level, Others had a relative abundance of 75.67–94.97%, which means that many species could not be determined. The determined dominant species which had a relative abundance of more than 1% were *Pediococcus acidilactici* (4.51% and 2.85%), *Weissella cibaria* (1.88% and 6.88%), and *Lactobacillus pentosus* (1.52% and 7.69%) in Se0 and Se3; *Pediococcus acidilactici* (4.68%), *Weissella cibaria* (2.10%), *Lactobacillus fermentum* (2.02%), and *Lactobacillus pentosus* (1.36%) in Se1; *Pediococcus acidilactici* (3.71%) and *Lactobacillus pentosus* (3.37%) in Se2; *Pediococcus acidilactici* (5.71%) and *Lactobacillus fermentum* (2.72%) in Se4; *Pediococcus acidilactici* (7.88% and 4.29%), *Lactobacillus pentosus* (2.18% and 5.16%), and *Escherichia coli* (1.83% and 2.34%) in Se5 and Se6; *Weissella cibaria* (19.19%), *Escherichia coli* (2.34%), and *Pediococcus acidilactici* (1.81%) in Se7; and *Weissella cibaria* (2.22%), *Clostridium sardiniense* (1.10%), and *Clostridium perfringens* (1.10%) in Se8.

#### 3.3.3. Alpha Diversity Analysis in Different HPM7 Silages

Alpha diversity analysis can reflect the richness and diversity of microbial communities. The Shannon and Simpon indices can reflect the diversity of microbial communities, while the Chao 1 and Ace indices reflect community richness. There was no significant difference in the Simpson index and coverage between the groups (*p* > 0.05). Compared with the Se0 group, the Shannon index of the Se1 and Se2 groups were significantly increased (*p* < 0.05), while the Chao1 index and ACE index of the Se1, Se2, Se3, Se5 and Se6 groups were significantly increased (*p* < 0.05) (Table 5).

**Figure 4:**
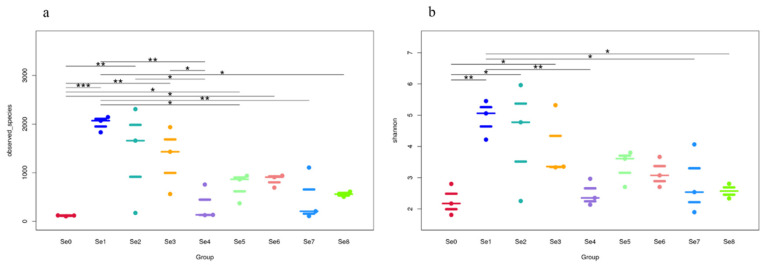
The beeswarm of observed species index (**a**) and Shannon index (**b**) in different HPM7 silages. Note: Se0 to Se8 represent selenium fertilizer solubility levels of 0, 0.50 mg/kg, 1.00 mg/kg, 2.00 mg/kg, 5.00 mg/kg, 10.00 mg/kg, 20.00 mg/kg, 30.00 mg/kg, and 40.00 mg/kg, respectively. * means *p* < 0.05, ** means *p* < 0.01, *** means *p* < 0.001.

The beeswarm of observed species index (Figure 4a) showed the scatter distribution of the total number of species among different groups of all samples, namely richness and the Shannon index (Figure 4b), and the differences in species diversity and evenness among different samples (* *p* < 0.05, ** *p* < 0.01, *** *p* < 0.001). There was no significant difference between the Se3–Se4, Se2–Se4, and Se0–Se5 groups (*p* > 0.05), and there was a significant difference in the number of species measured between the Se0–Se1, Se0–Se2, and Se0–Se3 groups (*p* < 0.05) and in the Shannon index between the Se0–Se1, Se1–Se4, and Se0–Se3 groups (*p* < 0.05) (Figure 4).

#### 3.3.4. Beta Diversity Analysis in Different HPM7 Silages

**Figure 5:**
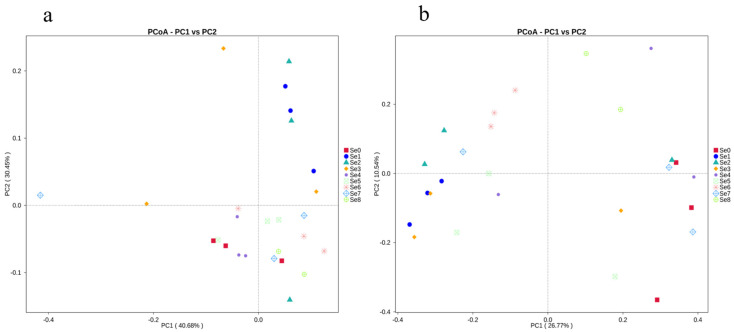
Based on weighted Unifrac (**a**) distance and unweighted Unifrac (**b**) of PCoA analysis. Note: Se0 to Se8 represent selenium fertilizer solubility levels of 0, 0.50 mg/kg, 1.00 mg/kg, 2.00 mg/kg, 5.00 mg/kg, 10.00 mg/kg, 20.00 mg/kg, 30.00 mg/kg, and 40.00 mg/kg, respectively.

To visualize the similarity and dissimilarity in postmortem bacterial composition among different HPM7 silages, a principal coordinate analysis (PCoA) was used to demonstrate the decomposing pattern in two-dimensional space based on the unweighted and weighted UniFrac distances. Principal coordinate 1 (PCo1) and PCo2 (unweighted were 26.77% and 10.54% of variance explained, respectively, while weighted were 40.68% and 30.45% of variance explained, respectively) axes showed the microbial communities of different silages (Figure 5).

The distance between the Se0 and Se4~Se8 groups was similar, indicating that the species composition between the two groups was similar. The distance between the Se1, Se2, and Se3 groups and the other groups was relatively different, indicating that the microbial community composition of these two groups is significantly different from the other groups.

**Figure 6:**
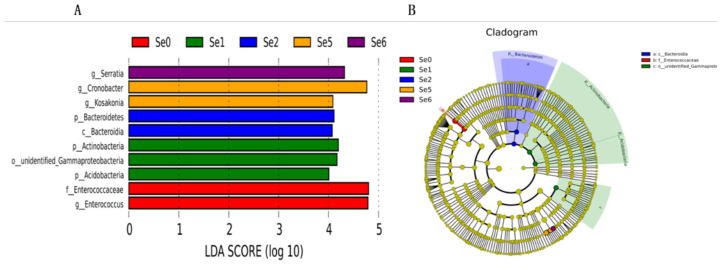
LEfSe analysis. Note: (**A**) LDA SCORE; (**B**) Cladogram. Se0, Se1, Se2, Se5, and Se6 represent selenium fertilizer solubility levels of 0, 0.50 mg/kg, 1.00 mg/kg, 10.00 mg/kg, and 20.00 mg/kg, respectively.

Considering that the DESeq2 analysis was unable to determine the dominant flora among the groups, a Lefse analysis with an LDA score of 4 was utilized, as shown in Figure 6. The Lefse analysis revealed a total of 10 bacteria that were identified as potential biomarker bacteria (Figure 6A). Specifically, the Se0 group had two significantly different bacteria (*f_Enterococcaceae*, *g_Enterococcus*), the Se1 group had three significantly different bacteria (*p_Actinobacteria*, *o_*unidentified*_Gammaproteobacteria*, *p_Acidobacteria*), the Se2 group had two significantly different bacteria (*p_Bacterioidetes*, *c_Bacteroidia*), the Se5 group had two significantly different bacteria (*g_Cronobacter*, *g_Kosakonia*), and the Se6 group had one significantly different bacterium (*g_Serratia*). Notably, the Se1 group exhibited the highest number of significant biomarkers compared to the other groups in this study (Figure 6B).

**Figure 7:**
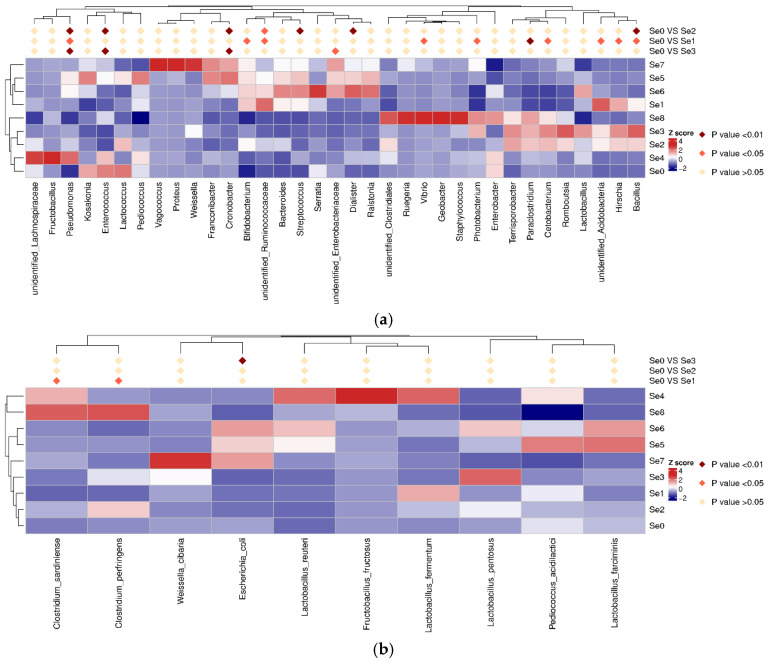
MetaStat analysis at genus level (**a**) and at species level (**b**). Note: Se0 to Se8 represent selenium fertilizer solubility levels of 0, 0.50 mg/kg, 1.00 mg/kg, 2.00 mg/kg, 5.00 mg/kg, 10.00 mg/kg, 20.00 mg/kg, 30.00 mg/kg, and 40.00 mg/kg, respectively.

A MetaStat analysis was performed to identify specific microorganisms at the genus level (Figure 7a) and at the species level (Figure 7b) with significant differences in different HPM7 silages. The genus, including *Enterococcus* and *Lactococcus* in the Se0 group; unidentified *Ruminococcaceae* and unidentified *Acidobacteria* in the Se1 group; *Romboutsia* and Bacillus in the Se3 group; unidentified *Lachnospiraceae*, *Fructobacillus*, and *Pseudomonas* in the Se4 group; *Kosakonia*, *Pediococcus*, *Franconibacter*, and *Cronobacte* in the Se5 group; *Serratia* in the Se6 group; *Vagococcus*, *Proteus*, and *Weissella* in the Se7 group; and unidentified *Clostridiales*, *Ruegeria*, *Vibrio*, *Geobacter*, and *Staphylococcu* in the Se8 group, were much higher than in the other groups. The species, including *Lactobacillus pentosu* in the Se3 group; *Lactobacillus reuteri*, *Fructobacillus fructosus,* and *Lactobacillus fermentum* in the Se4 group; *Pediococcus acidilactici* and *Lactobacillus farciminis* in the Se5 group; *Weissella cibari* in the Se7 group; and *Clostridium sardiniense* and *Clostridium perfringens* in the Se8 group, were much higher than other groups. Comparing the bacteria (*Fructobacillus*, *Enterococcus*, *Cronobacter*, unidentified *Enterobacteriaceae*, *Streptococcus*, *Dialister*, *Bacillus*) between the Se0 group and the Se2 group, bacteria (*Fructobacillus*, unidentified *Enterobacteriaceae*, *Vibrio*, *Photobacterium*, *Paraclostridium*, unidentified *Acidobacteria*, *Hirschia*, *Bacillus*) between the Se0 group and the Se1 group, bacteria (*Fructobacillus*, *Enterococcus*, *Cronobacter*, unidentified *Enterobacteriaceae*) between the Se0 group and Se3 group showed significant differences (*p* < 0.01 and *p* < 0.05) at the genus level. *Escherichia coli* between the Se0 group and the Se3 group and *Clostridium sardiniense* and *Clostridium perfringens* between the Se0 group and the Se1 group showed significant differences (*p* < 0.01 and *p* < 0.05) at the species level.

#### 3.3.5. Functional Prediction in Different HPM7 Silages

**Figure 8:**
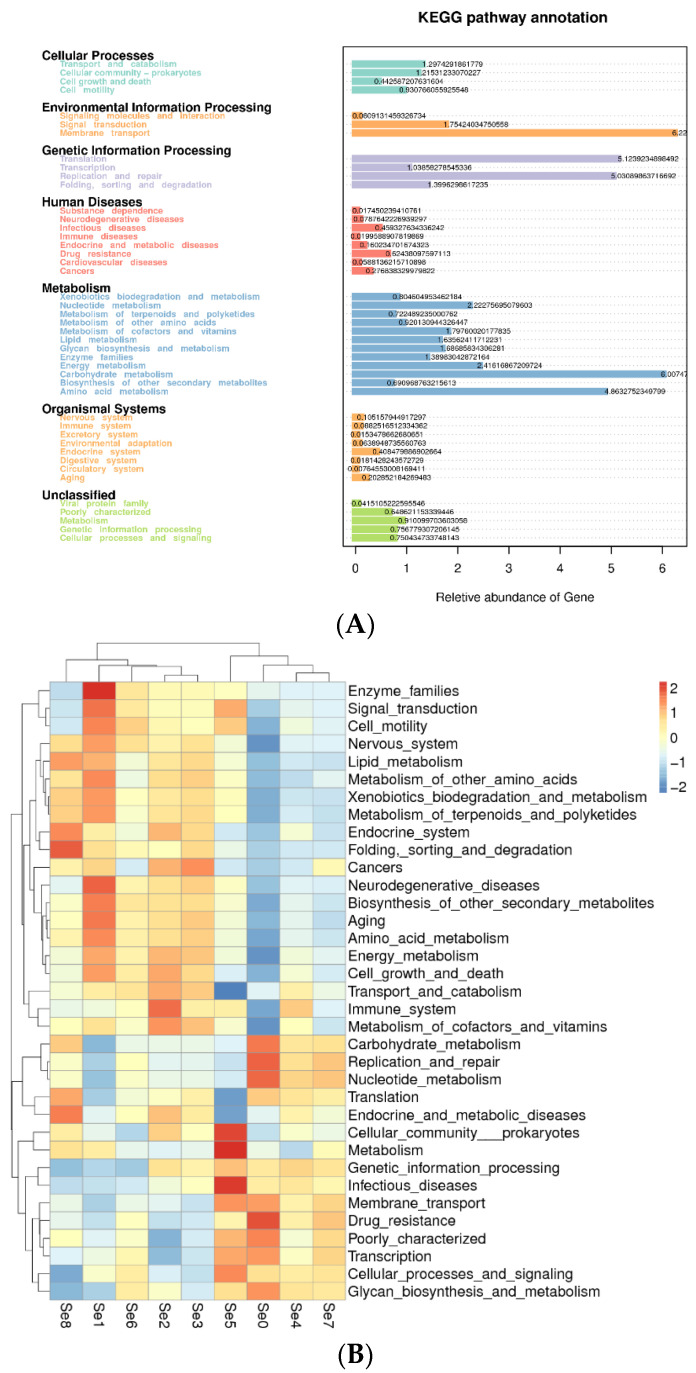
Functional prediction analysis. Note: (**A**) KEGG pathway analysis; (**B**) heatmap of functional prediction. Se0 to Se8 represent selenium fertilizer solubility levels of 0, 0.50 mg/kg, 1.00 mg/kg, 2.00 mg/kg, 5.00 mg/kg, 10.00 mg/kg, 20.00 mg/kg, 30.00 mg/kg, and 40.00 mg/kg, respectively.

To further understand the microbial function, the Tax4Fun method was used to predict the microbial function of silage. The second level of KEGG pathways is shown in the clustering heatmap (Figure 8B). The top-five most abundant pathways in all groups for metabolism were membrane transport (6.22), carbohydrate metabolism (6.01), translation (5.12), replication and repair (5.05), and amino acid metabolism (4.86) (Figure 8A). Certain pathways, including human diseases and genetic information processing in the Se0 group; cellular processes, organismal systems, and metabolism in the Se1, Se2, and Se3 group; and environmental information processing in the Se5 group, were much higher than in other groups. In contrast, cellular processes, organismal systems, and metabolism in Se0 and Se7 were much lower than in other groups.

#### 3.3.6. Correlation Analysis in Different HPM7 Silages

**Figure 9:**
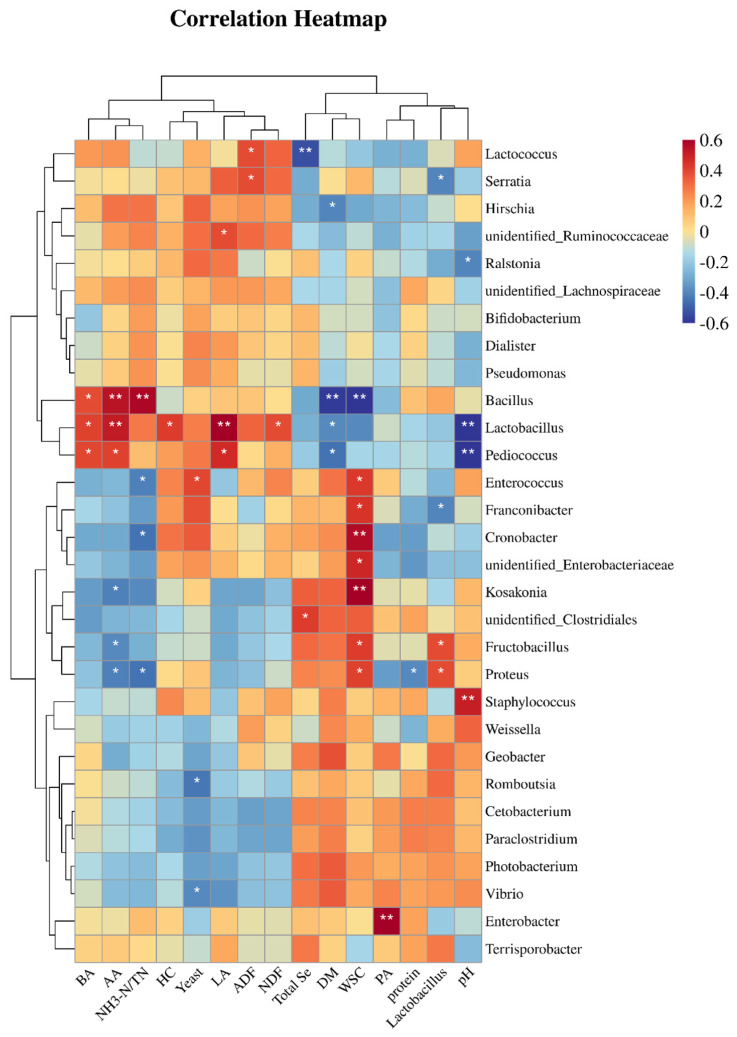
The correlation analysis between the microbe and the quality index of silage. Note: * means *p* ≤ 0.05, ** means *p* ≤ 0.01.

To study the correlation between the microbe and the quality index of silage fermentation, the Sperman correlation coefficient was used to analyze the microbe and the quality index of silage (Figure 9). In silage, the DM content was significantly negatively correlated with *Bacillus*, *Lactobacillus*, *Pediococcus*, and *Hirschia* (*p* < 0.01, *p* < 0.05); the NDF and HC contents were significantly positively correlated with *Lactobacillus* (*p* < 0.05); the protein content was significantly negatively correlated with proteus (*p* < 0.05); the NH_3_-N/TN was significantly positively correlated with *Bacillus* (*p* < 0.01) and significantly negatively correlated with *Enterococcus*, *Cronobacter*, and *Proteus* (*p* < 0.05); the pH value was significantly positively correlated with *Staphylococcus* (*p* < 0.01) and negatively correlated with *Lactobacillus*, *Pediococcus*, and *Ralstonia* (*p* < 0.01, *p* < 0.05); the LA content was significantly positively correlated with *Lactobacillus*, *Pediococcus*, and unidentified-*Ruminococcaceae* (*p* < 0.01, *p* < 0.05); the AA content was significantly positively correlated with *Lactobacillus*, *Pediococcus*, and *Bacillus* (*p* < 0.01, *p* < 0.05) and negatively correlated with *Kosakonia*, *Fructobacillus*, and *Proteus* (*p* < 0.05); the PA content was significantly positively correlated with *Enterobacter* (*p* < 0.01); the BA content was significantly positively correlated with *Lactobacillus*, *Pediococcus*, and *Bacillus* (*p* < 0.05); and the total Se content was significantly negatively correlated with *Lactococcus* and *Hirschia* (*p* < 0.01). The most noteworthy finding is that the total Se content was significantly positively correlated with unidentified *Clostridiales* (*p* < 0.05) and negatively correlated with *Lactococcus* (*p* < 0.01).

## 4. Discussion

Although no studies have confirmed that Se is an essential element for plant growth, a substantial body of research has demonstrated its dual effects on plants. An appropriate dosage can stimulate plant growth and enhance nutritional quality [26,27], while excessive amounts may lead to toxic effects [28]. Se plays a crucial role in regulating the formation of antioxidants in plants, including glutathione peroxidase (GSH-PX), reduced glutathione (GSH), superoxide dismutase (SOD), catalase (CAT), peroxidase (POD), and thioredoxin reductase (TrxR) [29]. Adequate Se supplementation can elevate the antioxidant levels within the organism by enhancing the activity of Se-containing antioxidative enzymes and reducing concentrations of hydrogen peroxide (H_2_O_2_) and malondialdehyde (MDA) [30], thereby improving plant stress resistance. A suitable amount of Se also increases chlorophyll content, promotes photosynthesis, and enhances carbohydrate and protein metabolism in forage grasses, ultimately fostering plant growth and increasing dry matter yield [31,32]. Se toxicity occurs when its concentration in plants exceeds optimal levels; excessive Se induces oxidative stress, generates reactive oxygen species (ROS), disrupts sulfur metabolism, and alters protein structure and function, leading to metabolic abnormalities that hinder plant growth and development [33,34,35]. Research by Bai et al. indicated that applying more than 20.00 mg/kg of Se to soil significantly inhibited alfalfa growth [11]. Similarly, another study found that exceeding 20.00 mg/kg application resulted in inhibited ryegrass growth alongside decreased GSH-Px activity and root vitality [36]. The findings suggest that HPM7 exhibits strong tolerance to Se; as the concentration of Se fertilizer increased from 0.50 to 40.00 mg/kg, the Se content within HPM7 rose significantly—ranging from 1.65 mg/kg to 11.6 mg/kg across Se1~Se8 groups. However, seedlings did not survive at a concentration of 50.00 mg/kg Se added to soil in group Se9; this indicates that HPM7′s tolerance limit for Se fertilizer should not exceed this threshold. In this experiment, various nutritional indices for hybrid *Pennisetum* exhibited changes: with increasing Se treatment amounts initially decreasing then subsequently increasing dry matter content, protein content rising before declining, while NDF content showed varying degrees of decrease—all potentially linked to differing treatment concentrations. These results indicate distinct impacts from varying concentrations of Se on HPM7′s photosynthesis as well as carbohydrate and protein metabolism.

It was worth noting that Se fertilizer may promote soluble carbohydrate metabolism in HPM7. There were reports that Se may be involved in carbohydrate metabolism in plants. Zeng et al. reported that the anti-stress, anti-oxidation, active oxygen metabolism, and carbohydrate and amino acid metabolism of natural Se-enriched rice was higher than that of non-Se rice [37]. Tan et al. found that the combined application of sucrose and Se could effectively promote vitamin C, sucrose, and fructose contents, especially the Se content [38]. In our experiment, the water-soluble carbohydrate content also exhibited an increase with the application of a low concentration of Se fertilizers (Se1, Se2, and Se3 groups). The metabolic pathway annotation result showed that in the WSC metabolism was in second place. The result about table grapes proved that acid invertase played an important role in the process of carbohydrate accumulation in barriers [39].

Differences in DM, CP, ADF, and hemicellulose contents between treatments after silage were attributed to differences between raw materials, indicating that the fermentation of forage with different Se content had no significant effects on these parameters. However, it had more pronounced effects on the WSC and NDF. WSC levels in raw materials treated with low concentration of Se were significantly higher than those in other groups but decreased significantly after fermentation. And it also appears in NDF, which is significantly reduced at first and significantly increased after fermentation in the Se1, Se2, and Se3 groups. And the pH of Se3, Se4, and Se6 were significantly lower than the Se0’s. Se may improve the fermentation quality of HPM7 silage through two aspects: on the one hand, it may promote the reproduction and metabolism of anaerobic bacteria (LAB) and promote LA fermentation. On the other hand, it may be to increase the proportion of fiber-degrading microorganisms, so as to provide more substrates for the activities of LAB. Axley et al. reported that Se plays an important role in the metabolism of anaerobic bacteria [40]. It can bind specifically to bacterial transport RNA and bacterial proteins [40]. At the same time, Se can promote carbohydrate metabolism in LAB fermentation [41]. In addition, the application of a low concentration of Se fertilizer (Se1, Se2, and Se3 groups) enhanced the microorganism relative abundance of HPM7 silages. Through microbiome analysis, it was found that *Actinobacteria*, *Chloroflexi*, and *Acidobacteria* in the Se1, Se2, and Se3 groups and *Bactroidetes* and *Gemmatimonadete* in the Se1 and Se2 groups were significantly higher than in Se0 after silage. Firmicutes can degrade macromolecular substances, such as cellulose, starch, protein, etc. Furthermore, *Firmicutes* and *Proteobacteria* can degrade fibrous substances at the same time, providing more substrates for microbial activities [42]. *Proteobacteria* is a class of Gram-negative bacteria, including many pathogenic bacteria, such as *Escherichia coli*, *Salmonella*, etc., which can compete with LAB for substrate and lead to the decrease in CP content and the increase in ammonia nitrogen content. The results of this experiment were consistent with this theory. *Firmicutes* and *Proteobacteria* were the dominant bacteria in HPM7 silage. Compared with the control, with the increase in Se content, the relative abundance of *Proteobacteria* decreased by 1.84%~14.11%.

Currently, there is a lack of relevant research literature on this topic, and certain phenomena remain unobserved. For instance, before silage preparation, the concentrations of DM, CP, NDF, and ADF in HPM7 silage exhibited significant variations under different levels of Se fertilizer treatment. Correlation analysis results suggest a potential strong relationship with the plant’s microbial flora; however, the specific mechanisms underlying microbial interactions have not been documented in the existing literature and warrant further investigation.

## 5. Conclusions

This study investigated, modulated, and analyzed the impact of different solubilities of an Se fertilizer of HPM7 on silage quality and microbial diversity. According to the results, the application of the Se fertilizer could enhance the Se content in HPM7, and its seedling survival rate decreases as the application concentration of Se increases. A certain amount of Se fertilizer may promote soluble carbohydrate metabolism in HPM7 and had more pronounced effects on WSC and NDF. In total, 0.50~2.00 mg/kg of the Se fertilizer could enhance the fermentation quality and relative microorganism abundance of HPM7 silages. Additionally, it remains an open question whether feeding animals with Se-enriched HPM7 grass can enhance the Se content in animal products, which is another area that our project team intends to explore further.

## Figures and Tables

**Table 1:** Characteristics of raw materials.

Item	Groups									
	Se0	Se1	Se2	Se3	Se4	Se5	Se6	Se7	Se8	Se9
Selenium (mg/kg)	0.32 ± 0.03 i	1.65 ± 0.02 h	2.81 ± 0.02 g	3.78 ± 0.01 f	7.84 ± 0.03 e	9.36 ± 0.08 d	9.68 ± 0.15 c	10.0 ± 60.23 b	11.60 ± 0.28 a	NSC
Dry matter (%)	28.67 ± 1.32 d	27.75 ± 0.43 de	25.75 ± 0.49 f	26.14 ± 1.09 ef	30.99 ± 1.92 c	30.85 ± 0.81 c	34.08 ± 1.01 b	37.42 ± 1.30 a	37.32 ± 0.14 a	NSC
Crude protein (% DM)	6.26 ± 0.02 bcd	6.74 ± 0.15 a	6.63 ± 0.13 ab	6.38 ± 0.10 abc	6.27 ± 0.08 bcd	6.24 ± 0.07 bcd	6.17 ± 0.23 cd	5.95 ± 0.21 d	5.88 ± 0.49 d	NSC
Water-soluble carbohydrates (% DM)	11.98 ± 0.15 c	12.93 ± 0.36 bc	13.93 ± 0.66 ab	14.72 ± 0.53 a	12.94 ± 0.41 bc	14.06 ± 0.84 a	12.51 ± 0.60 c	12.43 ± 0.88 c	12.16 ± 0.64 c	NSC
Neutral detergent fiber (% DM)	61.12 ± 0.34 a	59.28 ± 1.17 bc	58.86 ± 2.09 bc	58.46 ± 0.03 c	59.15 ± 0.36 bc	60.46 ± 0.21 ab	56.61 ± 1.51 d	56.30 ± 0.34 d	60.57 ± 0.62 ab	NSC
Acid detergent fiber (% DM)	31.91 ± 2.64 ab	34.28 ± 6.59 ab	32.36 ± 3.89 ab	37.53 ± 8.82 a	28.24 ± 0.75 b	28.55 ± 0.20 b	28.33 ± 1.59 b	27.63 ± 0.63 b	29.22 ± 0.32 b	NSC
Hemi-cellulose (% DM)	29.21 ± 2.30 a	24.99 ± 5.42 ab	26.49 ± 5.98 ab	20.93 ± 8.79 b	30.91 ± 0.38 a	31.91 ± 0.01 a	28.27 ± 0.08 ab	28.68 ± 0.97 a	31.35 ± 0.93 a	NSC
Ash (% DM)	7.65 ± 0.31	7.73 ± 0.54	7.89 ± 0.01	7.70 ± 0.34	7.68 ± 0.18	7.67 ± 0.04	7.44 ± 0.54	7.40 ± 0.23	7.38 ± 0.02	NSC
Ether extract (% DM)	5.31 ± 0.06 ab	5.69 ± 0.11 ab	5.79 ± 0.16 a	5.57 ± 0.20 ab	5.40 ± 0.31 ab	5.30 ± 0.02 ab	5.49 ± 0.11 ab	5.23 ± 0.54 ab	5.06 ± 0.49 b	NSC
Lactic acid bacteria (lg cfu/g FM)	4.25 ± 0.32	4.18 ± 0.17	4.37 ± 0.24	4.31 ± 0.13	4.24 ± 0.28	4.15 ± 0.43	4.40 ± 0.19	4.22 ± 0.26	4.20 ± 0.25	NSC
Aerobic bacteria (lg cfu/g FM)	6.50 ± 0.47	6.45 ± 0.88	6.38 ± 0.42	6.48 ± 0.60	6.39 ± 0.23	6.42 ± 0.54	6.23 ± 0.33	6.40 ± 0.36	6.39 ± 0.14	NSC
Yeast (lg cfu/g FM)	5.41 ± 0.18	5.20 ± 0.24	5.35 ± 0.17	5.32 ± 0.53	5.40 ± 0.22	5.67 ± 0.45	5.29 ± 0.30	5.31 ± 0.13	5.26 ± 0.44	NSC
Mold (lg cfu/g FM)	3.05 ± 0.41	3.23 ± 0.31	3.07 ± 0.09	3.16 ± 0.35	3.01 ± 0.26	3.11 ± 0.24	3.22 ± 0.07	3.25 ± 0.28	3.10 ± 0.17	NSC

Note: Data ± SD are means of three samples. None of the seven replicates in the Se9 group survived and therefore had no data. DM, dry matter; Se0 to Se9 represented selenium fertilizer solubility levels of 0, 0.50 mg/kg, 1.00 mg/kg, 2.00 mg/kg, 5.00 mg/kg, 10.00 mg/kg, 20.00 mg/kg, 30.00 mg/kg, 40.00 mg/kg, and 50.00 mg/kg, respectively. NSC, no samples were collected. Different lowercase letters in peer data indicate significant differences between different treatments (*p* < 0.05).

**Table 2:** The chemical composition and fermentation quality of the silages.

Item	Groups								
	Se0	Se1	Se2	Se3	Se4	Se5	Se6	Se7	Se8
Dry matter (%)	30.31 ± 3.48 c	28.03 ± 2.39 cd	25.94 ± 0.35 d	26.18 ± 0.45 d	31.27 ± 0.94 bc	31.53 ± 0.29 bc	34.56 ± 2.44 ab	37.06 ± 1.81 a	38 ± 2.17 a
Crude protein (% DM)	6.54 ± 0.03 bc	6.79 ± 0.05 a	6.66 ± 0.13 ab	6.53 ± 0.06 bcd	6.26 ± 0.08 e	6.27 ± 0.17 e	6.42 ± 0.02 cd	5.99 ± 0.06 f	6.38 ± 0.08 de
Water-soluble carbohydrates (% DM)	3.71 ± 0.23 b	2.49 ± 0.14 c	2.01 ± 0.14 c	1.62 ± 0.15 c	4.63 ± 0.41 ab	5.01 ± 0.36 a	5.09 ± 1.47 a	5.04 ± 0.16 a	4.45 ± 0.21 ab
Neutral detergent fiber (% DM)	61.51 ± 0.16 a	61.57 ± 0.77 a	60.75 ± 1.85 a	58.84 ± 1.57 ab	61.62 ± 0.7 a	59.98 ± 0.57 ab	58.58 ± 1.18 ab	59.24 ± 3.69 ab	56.74 ± 2.39 b
Acid detergent fiber (% DM)	34.51 ± 0.68 ab	35.81 ± 0.62 a	32.63 ± 2.24 b	34.32 ± 2.25 ab	35.77 ± 0.58 a	35.59 ± 1.12 a	33.93 ± 0.91 ab	34.1 ± 1.78 ab	32.41 ± 0.58 b
Hemi-cellulose (% DM)	27.00 ± 0.84 ab	25.76 ± 0.15 ab	28.11 ± 1.09 a	24.52 ± 2.82 b	25.85 ± 1.28 ab	24.39 ± 1.5 b	24.65 ± 0.46 b	25.14 ± 1.91 b	24.33 ± 1.81 b
pH value	5.58 ± 0.01 ab	5.38 ± 0.08 bc	5.35 ± 0.04 bc	5.28 ± 0.18 c	5.30 ± 0.19 c	5.36 ± 0.04 bc	5.20 ± 0.24 c	5.74 ± 0.03 a	5.78 ± 0.08 a
Lactic acid (g/kg DM)	10.68 ± 1.25 b	12.69 ± 1.63 a	11.85 ± 1.33 a	11.1 ± 1.37 ab	10.79 ± 1.65 b	11.64 ± 4.5 ab	11.57 ± 1.61 ab	8.14 ± 1.19 c	7.78 ± 1.01 c
Acetic acid (g/kg DM)	0.12 ± 0.01 ab	0.15 ± 0.03 a	0.13 ± 0.01 a	0.15 ± 0.05 a	0.07 ± 0.02 cd	0.09 ± 0.04 bc	0.05 ± 0.01 de	0.03 ± 0.01 e	0.05 ± 0 de
Propionic acid (g/kg DM)	0.03 ± 0.01 abc	0.02 ± 0.01 abc	0.03 ± 0.01 abc	0.03 ± 0.02 ab	0.03 ± 0 abc	0.02 ± 0.01 bc	0.03 ± 0.01 a	0.02 ± 0.01 c	0.03 ± 0.01 ab
Butyric acid (g/kg DM)	0.01 ± 0.01 ab	0.01 ± 0.01 ab	0.01 ± 0.01 ab	0.01 ± 0.00 a	0.01 ± 0.01 abc	0.01 ± 0.00 c	0.01 ± 0.00 bc	0.00 ± 0.00 c	0.00 ± 0.00 c
Ammonia N/total N (g/kg DM)	1.55 ± 0.17	1.56 ± 0.18	1.58 ± 0.27	1.49 ± 0.23	1.39 ± 0.09	1.33 ± 0.01	1.38 ± 0.02	1.47 ± 0.12	1.56 ± 0.03
Lactic acid bacteria (lg cfu/g FM)	8.23 ± 0.22 c	8.77 ± 0.08 ab	8.85 ± 0.14 a	8.59 ± 0.05 abc	8.50 ± 0.37 abc	8.66 ± 0.11 abc	8.37 ± 0.34 bc	8.82 ± 0.31 a	8.88 ± 0.2 a
Yeast (lg cfu/g FM)	6.18 ± 0.2 ab	6.29 ± 0.2 ab	6.25 ± 0.31 ab	6.28 ± 0.15 ab	6.35 ± 0.47 ab	6.73 ± 0.39 a	6.28 ± 0.28 ab	5.92 ± 0.57 b	5.92 ± 0.58 b

Note: Data ± SD are means of three samples. None of the seven replicates in Se9 group survived and therefore had no data. DM, dry matter; Se0 to Se9 represent selenium fertilizer solubility levels of 0, 0.50 mg/kg, 1.00 mg/kg, 2.00 mg/kg, 5.00 mg/kg, 10.00 mg/kg, 20.00 mg/kg, 30.00 mg/kg, 40.00 mg/kg, and 50.00 mg/kg, respectively. NSC, no samples were collected. Different lowercase letters in peer data indicate significant differences between different treatments (*p* < 0.05).

**Table 3:** Relative abundance of microorganisms based on phylum level (%).

Items	Groups								
	Se0	Se1	Se2	Se3	Se4	Se5	Se6	Se7	Se8
*Proteobacteria*	69.04 ± 7.35	65.74 ± 4.54	65.27 ± 6.02	59.18 ± 14.24	67.56 ± 2.88	67.77 ± 3.23	73.29 ± 7.69	61.6 ± 19.94	73.5 ± 6.3
*Firmicutes*	30.1 ± 7.25	21.01 ± 1.43	21.34 ± 0.79	28.19 ± 11.52	30.47 ± 2.44	28.82 ± 3.7	23.27 ± 7.57	35.53 ± 21.32	21.68 ± 5.56
*Fusobacteria*	0.77 ± 0.28	0.00 ± 0.00	2.49 ± 4.31	3.04 ± 5.27	1.06 ± 0.92	0.6 ± 1.03	0.00 ± 0.00	0.76 ± 0.66	2.07 ± 0.47
*Bacteroidetes*	0.01 ± 0.01 c	2.63 ± 0.65 ab	3.08 ± 2.83 a	1.98 ± 1.93 bc	0.24 ± 0.42 bc	0.63 ± 0.71 bc	1.1 ± 0.45 bc	0.63 ± 1.08 bc	0.16 ± 0.03 bc
*Actinobacteria*	0.05 ± 0.03 c	3.29 ± 0.54 a	2.79 ± 2.14 ab	2.51 ± 1.06 ab	0.28 ± 0.42 c	1.05 ± 0.4 bc	0.99 ± 0.06 bc	0.7 ± 0.98 c	1.36 ± 0.03 bc
*Chloroflexi*	0.00 ± 0.00 c	2.04 ± 0.57 a	1.49 ± 1.31 ab	1.40 ± 1.35 ab	0.12 ± 0.2 bc	0.28 ± 0.19 bc	0.38 ± 0.09 bc	0.17 ± 0.24 bc	0.15 ± 0.01 bc
*Acidobacteria*	0.00 ± 0.00 c	2.14 ± 0.53 a	1.07 ± 1.06 ab	1.29 ± 0.97 ab	0.06 ± 0.11 c	0.32 ± 0.14 bc	0.39 ± 0.24 bc	0.12 ± 0.2 c	0.35 ± 0.01 bc
*Gemmatimonadetes*	0.00 ± 0.00 c	1.22 ± 0.29 a	0.99 ± 0.91 ab	0.89 ± 0.81 abc	0.06 ± 0.11 cd	0.2 ± 0.12 cbd	0.18 ± 0.04 cbd	0.10 ± 0.17 cd	0.09 ± 0.02 cd
*Cyanobacteria*	0.01 ± 0.02 b	0.02 ± 0.01 b	0.08 ± 0.06 b	0.1 ± 0.13 b	0.01 ± 0.01 b	0.01 ± 0.01 b	0.02 ± 0.03 b	0.18 ± 0.3 ab	0.32 ± 0.06 a
*Thaumarchaeota*	0.00 ± 0.00 b	0.08 ± 0.05 ab	0.06 ± 0.08 b	0.22 ± 0.23 a	0.01 ± 0.02 b	0.02 ± 0.02 b	0.01 ± 0.01 b	0.01 ± 0.02 b	0.00 ± 0.00 b
Others	0.02 ± 0.02 b	1.81 ± 0.52 a	1.35 ± 0.94 a	1.21 ± 0.72 a	0.12 ± 0.13 b	0.31 ± 0.13 b	0.36 ± 0.04 b	0.2 ± 0.28 b	0.33 ± 0.09 b

Note: Data ± SD are means of three samples. Se0 to Se8 represent selenium fertilizer solubility levels of 0, 0.50 mg/kg, 1.00 mg/kg, 2.00 mg/kg, 5.00 mg/kg, 10.00 mg/kg, 20.00 mg/kg, 30.00 mg/kg, and 40.00 mg/kg, respectively. Different lowercase letters in peer data indicate significant differences between different treatments (*p* < 0.05).

**Table 4:** Relative abundance of microorganisms based on genus level (%).

Items	Groups								
	Se0	Se1	Se2	Se3	Se4	Se5	Se6	Se7	Se8
*Enterobacter*	61.52 ± 12.01 ab	51.12 ± 6.62 ab	53.41 ± 13.26 ab	48.6 ± 14.57 ab	61.58 ± 4.9 ab	46.61 ± 8.44 ab	56.63 ± 12.81 ab	43.05 ± 14.69 b	66.79 ± 7.12 a
*Weissella*	1.89 ± 1.39 c	2.14 ± 2.82 c	0.73 ± 0.68 c	6.89 ± 1.65 b	0.80 ± 0.21 c	0.52 ± 0.52 c	1.28 ± 1.05 c	19.23 ± 2.46 a	2.23 ± 3.03 c
*Enterococcus*	17.85 ± 7.24 a	4.99 ± 2.35 bc	4.48 ± 1.81 c	4.32 ± 4.33 c	14.71 ± 6.06 ab	11.52 ± 3.97 abc	5.69 ± 1.53 bc	9.28 ± 7.48 abc	10.01 ± 5.89 abc
*Lactobacillus*	2.76 ± 1.00	3.85 ± 2.09	4.41 ± 3.55	8.24 ± 2.99	4.51 ± 3.17	3.95 ± 2.26	7.88 ± 7.48	1.06 ± 0.73	0.55 ± 0.5
*Cronobacter*	2.09 ± 1.18 c	0.48 ± 0.13 c	0.33 ± 0.36 c	0.34 ± 0.12 c	2.56 ± 1.38 c	12.43 ± 7.56 a	4.91 ± 3.33 bc	10.26 ± 5.34 ab	0.1 ± 0.06 c
*Pediococcus*	4.65 ± 2.25 abc	4.82 ± 3.8 abc	3.86 ± 1.14 abc	2.93 ± 1.75 bc	5.96 ± 2.38 ab	8.25 ± 4.44 a	4.47 ± 2.81 abc	1.9 ± 1.52 bc	0.32 ± 0.16 bc
*Cetobacterium*	0.77 ± 0.28	0.00 ± 0.00	2.49 ± 4.31	3.04 ± 5.27	1.06 ± 0.92	0.6 ± 1.03	0.00 ± 0.00	0.76 ± 0.66	2.07 ± 0.47
*Staphylococcus*	0.47 ± 0.34 b	0.3 ± 0.18 b	0.18 ± 0.17 b	0.66 ± 0.54 b	0.63 ± 0.31 b	0.19 ± 0.04 b	0.15 ± 0.11 b	0.4 ± 0.27 b	3.22 ± 4.09 a
*Paraclostridium*	0.56 ± 0.07	0.00 ± 0.00	1.83 ± 3.16	2.01 ± 3.48	0.84 ± 0.74	0.26 ± 0.46	0.00 ± 0.00	0.41 ± 0.37	2.14 ± 0.25
*Kosakonia*	2.57 ± 2.98 ab	0.21 ± 0.02 b	0.74 ± 0.71 ab	0.43 ± 0.19 ab	0.92 ± 0.68 ab	2.9 ± 1.25 a	1.41 ± 1.44 ab	1.49 ± 0.57 ab	0.71 ± 0.4 ab
Others	4.86 ± 2.33 c	32.08 ± 4.47 a	27.54 ± 16.37 a	22.54 ± 12.21 a	6.43 ± 4.67 c	12.77 ± 5.16 b	17.58 ± 3.84 b	12.15 ± 10.96 b	11.89 ± 0.66 b

Note: Data ± SD are means of three samples. Se0 to Se8 represent selenium fertilizer solubility levels of 0, 0.50 mg/kg, 1.00 mg/kg, 2.00 mg/kg, 5.00 mg/kg, 10.00 mg/kg, 20.00 mg/kg, 30.00 mg/kg, and 40.00 mg/kg, respectively. Different lowercase letters in peer data indicate significant differences between different treatments (*p* < 0.05).

**Table 5:** Effect of selenium on microbial alpha diversity of HPM7 silage.

Groups	Items				
	Shannon	Simpson	chao1	ACE	Coverage
Se0	2.26 ± 0.5 c	0.58 ± 0.14	136.86 ± 28.18 d	146.02 ± 29.3 d	1.00 ± 0.00
Se1	4.91 ± 0.63 a	0.73 ± 0.07	2248.24 ± 190.01 a	2331.72 ± 140.42 a	0.99 ± 0.00
Se2	4.34 ± 1.89 ab	0.69 ± 0.15	2222.85 ± 580.53 a	2289.28 ± 600.84 a	0.99 ± 0.01
Se3	4.00 ± 1.14 abc	0.71 ± 0.13	1877.02 ± 349.43 a	1921.51 ± 345.53 a	0.99 ± 0.01
Se4	2.49 ± 0.43 bc	0.6 ± 0.06	148.49 ± 5.43 d	156.94 ± 6.53 d	1.00 ± 0.00
Se5	3.37 ± 0.58 abc	0.74 ± 0.07	1032.39 ± 59.79 bc	1081.54 ± 44.56 bc	1.00 ± 0.00
Se6	3.15 ± 0.48 abc	0.65 ± 0.12	1115.95 ± 8.5 b	1216.02 ± 54.54 b	0.99 ± 0.01
Se7	2.84 ± 1.12 bc	0.67 ± 0.11	183.96 ± 50.01 d	184.47 ± 67.1 d	1.00 ± 0.01
Se8	2.58 ± 0.33 bc	0.54 ± 0.08	613.4 ± 78.87 cd	649.49 ± 96.71 cd	1.00 ± 0.00

Note: Data ± SD are means of three samples. Se0 to Se8 represent selenium fertilizer solubility levels of 0, 0.50 mg/kg, 1.00 mg/kg, 2.00 mg/kg, 5.00 mg/kg, 10.00 mg/kg, 20.00 mg/kg, 30.00 mg/kg, and 40.00 mg/kg, respectively. Different lowercase letters in peer data indicate significant differences between different treatments (*p* < 0.05).

## Data Availability

The original contributions presented in the study are included in the article, further inquiries can be directed to the corresponding authors.
